# m1A methylation modification patterns and metabolic characteristics in hepatocellular carcinoma

**DOI:** 10.1186/s12876-022-02160-w

**Published:** 2022-03-03

**Authors:** Chengcheng Tong, Wei Wang, Chiyi He

**Affiliations:** Department of Gastroenterology, Yijishan Hospital of Wannan Medical College, Wuhu, Anhui Province China

**Keywords:** m1A, Metabolism, Hepatocellular carcinoma, Prognosis

## Abstract

**Background:**

The dysregulation of RNA methylation has been demonstrated to contribute to tumorigenicity and progression in recent years. However, the alteration of N1-methyladenosine (m1A) methylation and its role in hepatocellular carcinoma (HCC) remain unclear.

**Methods:**

We systematically investigated the modification patterns of 10 m1A regulators in HCC samples and evaluated the metabolic characteristics of each pattern. A scoring system named the m1Ascore was developed using principal component analysis. The clinical value of the m1Ascore in risk stratification and drug screening was further explored.

**Results:**

Three m1A modification patterns with distinct metabolic characteristics were identified, corresponding to the metabolism-high, metabolism-intermediate and metabolism-excluded phenotypes. Patients were divided into high- or low-m1Ascore groups, and a significant survival difference was observed. External validation confirmed the prognostic value of the m1Ascore. A nomogram incorporating the m1Ascore and other clinicopathological factors was constructed and had good performance for predicting survival. Two agents, mitoxantrone and doxorubicin, were determined to be potential therapeutic drugs for the high-risk group.

**Conclusion:**

This study provided novel insights into m1A modification and metabolic heterogeneity in cancer, promoted risk stratification in the clinic from the perspective of m1A modification, and further guided individual treatment strategies.

**Supplementary Information:**

The online version contains supplementary material available at 10.1186/s12876-022-02160-w.

## Background

N1-methyladenosine (m1A), an RNA methylation pattern, is a dynamic RNA modification regulated by methyltransferases, demethylases and binding proteins [1]. The formation of m1A, which is catalyzed by methyltransferases, including TRMT10C, TRMT61B, TRMT6 and TRMT61A, is reversible by the ALKBH1 and ALKBH3 demethylases. YTHDF1, YTHDF2, YTHDF3 and YTHDC1 act as binding proteins that specifically recognize m1A sites and induce downstream effects [2]. The pioneering works by Dan et al. [3] and Li et al. [4] provided transcriptome-wide mapping of m1A and uncovered that m1A modification is enriched in the vicinity of the start codon, highlighting the roles of m1A in mRNA stability and translation. Today, it is recognized that the dynamic regulation of m1A in response to physiological stress and the dysregulated expression of m1A regulators are correlated with tumorigenesis and cancer recurrence [5, 6].

Metabolic reprogramming, as a hallmark of cancer, exists in nearly all cancer cells [7]. To support uncontrolled cell growth and proliferation, the metabolic manners of cancer cells are distinct from those of normal cells [8]. A classic metabolic reprogramming method is aerobic glycolysis, and cancer cells secrete lactate despite the aerobic environment present [9]. In addition, with a diversity of nutrients, including lipids, amid acids and nucleotides, found to take part in cancer metabolic reprogramming in addition to glucose [10], a consensus emerged based on the results from molecular-level clinical trials that showed different metabolic dependencies across cancer types [11], such as fatty acid metabolism in prostate cancer [12] and serine synthesis in breast cancer [13]. This difference, which is also recognized as metabolic heterogeneity, is mainly associated with the heterogeneity of stomatic alterations among cancer cells [14]. Drivers of oncogenesis vary in genomic alterations and subsequently affect different downstream pathways, finally reflecting heterogeneity in cancer metabolism phenotypes [15]. Studies reported by Haider et al. [16] and Sander et al. [17] provided sufficient evidence in the molecular dimension, which showed widespread transcriptional differences in metabolic-related genes between normal tissue and tumor tissue. Moreover, the identification of cancer metabolism phenotypes contributed to the reasonable stratification of patients in clinical practice with regard to the application of metabolic inhibitors and the individual selection of target therapies [18].

Although the effective classification of cancer metabolic characteristics remains a controversial issue to date, several pioneering works that focus on the expression pattern of metabolic genes have been performed in this frontier field and have identified distinct metabolic phenotypes with different sensitivities to metabolic inhibitors [19, 20]. Here, we explored m1A modification and its relationship with metabolic characteristics in hepatocellular carcinoma (HCC). Three m1A modification patterns with distinct metabolic characteristics were identified, corresponding to the metabolism-excluded, metabolism-high and metabolism-intermediate phenotypes, respectively. Clinical characteristics and prognosis showed significant differences among these phenotypes. Further, an m1A-related scoring system was developed and showed reliable performance in distinguishing drug sensitivities and stratifying risk in the clinic.

## Methods

### Data collection

Two RNA sequencing (RNA-seq) datasets were used in the current study: The Cancer Genome Atlas (TCGA, liver hepatocellular carcinoma (LIHC), https://www.cancer.gov) and International Cancer Genome Consortium (ICGC) (LIRI-JP, www.icgc.org). HCC patients with RNA-seq data (fragments per kilobase per million reads, FPKM) and corresponding clinical information were enrolled in March 2021.

### Statistical analysis

R 4.0.1 was applied to carry out all statistical tests. All tests were two-sided, and a *P* value < 0.05 was considered statistically significant.

Additional detailed protocols are provided in the Supplementary Methods.

## Results

### Landscape of genetic variations in m1A regulators in HCC

**Fig. 1:**
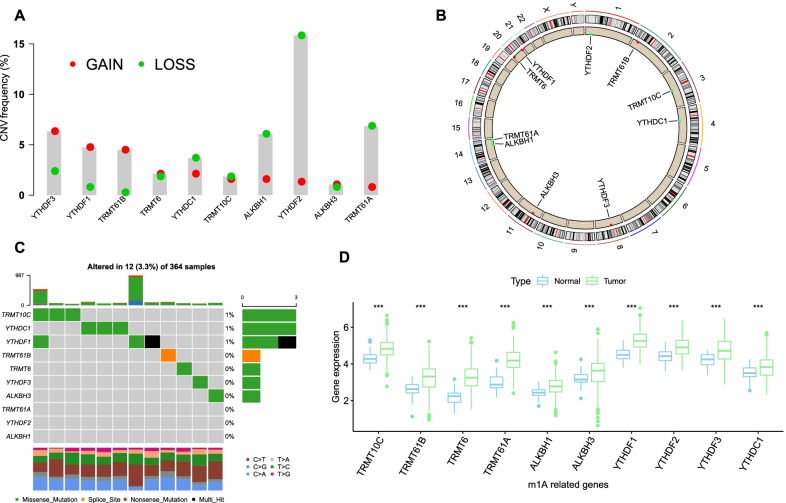
Genetic and expression landscapes of m^1^A regulators in HCC. **a** CNV frequency of m^1^A regulators in TCGA cohort. **b** The location of CNV of m^1^A regulators on chromosomes. **c** The mutation frequency of m^1^A regulators in TCGA cohort. Each column represents a patient. The upper barplot indicated the tumor mutation burden. The right barplot indicated the proportion of each mutation type. The lower barplot indicated fraction of conversions in each patient. **d** Expression levels of m^1^A regulators in HCC and normal tissues

There was a prevalent alteration of the copy number variation (CNV) in m1A regulators (Fig. 1a). Four regulators, YTHDF2, TRMT61A, ALKBH1 and YTHDC1, showed a majority of loss alterations, and three regulators, YTHDF3, YTHDF1 and TRMT61B, mainly had gain alterations. The locations of the CNV alterations of each m1A regulator on chromosomes are shown in Fig. 1b. Somatic mutations were also investigated in the TCGA cohort, and only a few samples showed mutations in m1A regulators, with a frequency of 3.33% (Fig. 1c). The mRNA expression levels of regulators were compared between normal and HCC samples, and a remarkable upregulation of each regulator was observed in HCC tissues (Fig. 1d, Additional file 1: Fig. S1a). Similar results were observed when comparatively estimating the expression of regulators in paired HCC and adjacent normal tissues (Additional file 1: Fig. S1b). The above results revealed significant heterogeneity at the genetic and epigenetic levels of m1A regulators between normal and HCC tissues.

### Identification of m1A methylation modification patterns

**Fig. 2:**
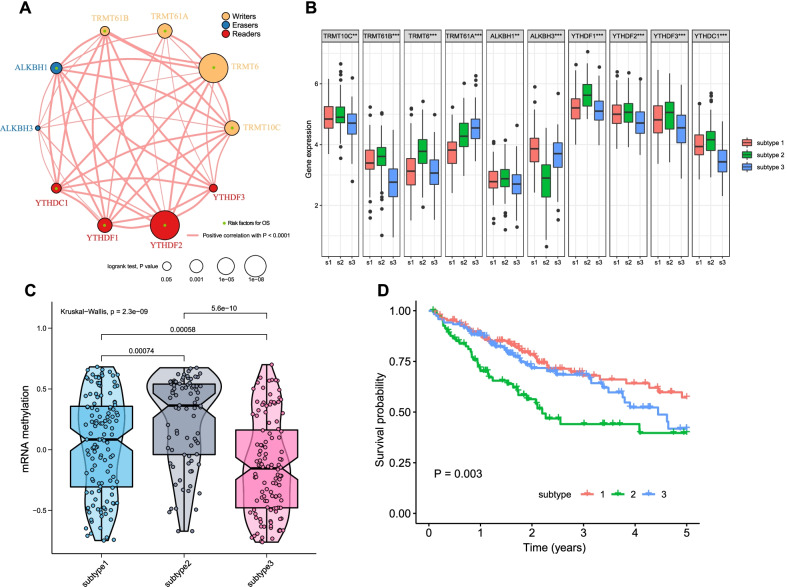
The m^1^A methylation modification patterns in HCC. **a** Network plot visualizing the interaction between m^1^A regulators in TCGA cohort. Each circle represents a m^1^A regulator gene and the color of circle represents the functional category. The circle size represents the impact of corresponding m^1^A regulator in survival and the lines between pairs circles indicate that there is an expression correlation between two regulators. Red line shows a positive correlation and thickness indicate the correlation strength. **b** Expression levels of m^1^A regulators in subtype 1–3. **c** Enrichment scores of mRNA methylation in subtype 1–3. **d** Overall survival of subtype 1–3 and the survival difference was evaluated by log-rank test

A network plot was used to visualize the connections and prognostic value of the regulators (Fig. 2a). A significantly positive correlation in expression was observed, and this correlation was prevalent despite the functional category of the m1A regulators. We found that TRMT10C, TRMT6, ALKBH1, YTHDF2, and YTHDF1 were positively correlated with the expression of other m1A regulators, and all of them were determined to be risk factors in univariate Cox analysis. In addition, none of the 10 m1A regulators was identified as a protective factor, revealing a similar prognostic effect among them.

Unsupervised cluster analysis was carried out using the expression data of m1A regulators, and three distinct modification patterns, which were named subtypes 1–3, were determined (Additional file 1: Fig. S2a-c). A total of 128 patients had subtype 1, 82 patients had subtype 2, and 120 patients had subtype 3. Subtype 2 had the highest m1A regulator expression levels compared with the other subtypes, while subtype 3 had the lowest expression levels (Fig. 2b). In accordance with the expression levels of m1A regulators in each subtype, a significant difference in the enrichment scores of mRNA methylation levels was observed among the three subtypes (Fig. 2c). Survival analysis showed that subtype 2 had a remarkably poorer prognosis, while subtype 1 and subtype 3 had better prognoses (Fig. 2d). These results revealed the modification heterogeneity of m1A regulators and distinct prognosis of HCC patients.

### Metabolic characteristics of the three m1A modification subtypes

**Fig. 3:**
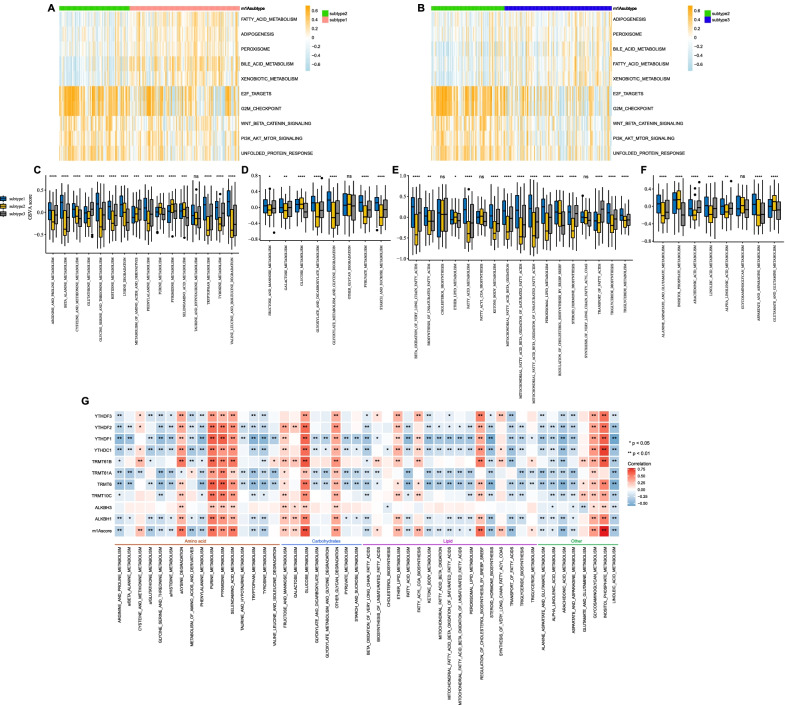
Biological characteristics involved in three m^1^A modification patterns. **a**, **b** Heatmap visualizing the activation states of biological pathways in subtype 1–3. The pathways achieved from the HALLMARK gene sets. **c-f** Gene set variation analysis enrichment scores of metabolic-related pathways in subtype 1–3. **c** Amino acid metabolism. **d** Carbohydrate metabolism. **e** Fatty acid metabolism. **f** Others metabolism. **g** The correlation plot

First, gene set variation analysis (GSVA) was performed in terms of the HALLMARK gene sets. As shown in Fig. 3a, b, the hallmarks related to metabolism, including fatty acid metabolism, xenobiotic metabolism and bile acid metabolism, were markedly downregulated in subtype 2. These results revealed low metabolic activity characterizing subtype 2, suggesting metabolic heterogeneity among the three subtypes. Thus, we further investigated the metabolic characteristics of each subtype. By estimating the GSVA enrichment scores of 49 metabolism-associated pathways, subtype 2 and subtype 1 had the lowest and highest enrichment scores, respectively, across the four major metabolic categories, respectively, while subtype 3 had modest values, strongly indicating that different m1A modification patterns were characterized by distinct metabolic activities (Fig. 3c–f, Additional file 1: Fig. S2d). Hence, subtype 1 was classified as a metabolism-high phenotype, corresponding to the hyperactive state in metabolism; subtype 2 was classified as a metabolism-excluded phenotype, corresponding to the hypoactive state; and subtype 3 was classified as a metabolism-intermediate phenotype.

We next explored the correlation between each m1A regulator and each metabolic pathway. Tumors with high activation of purine metabolism, pyrimidine metabolism, selenoamino acid metabolism, glucose metabolism, other glycan degradation and glycosaminoglycan metabolism showed a positive correlation with all regulators, while those with high activation of arginine and proline metabolism and linoleic acid metabolism were negatively correlated with all regulators. These results revealed that whether tumors with high expression of an m1A regulator showed high activation of a metabolic pathway was actually determined by the specific m1A regulator and metabolic pathway (Fig. 3g).

### Development of an m1A scoring system

270 overlapping m1A subtype-related genes were extracted from the different expression genes (DEGs) (Additional file 1: Fig. S3a). Unsupervised cluster analysis was performed again based on these 270 DEGs to classify patients into three genomic patterns (m1A gene clusters 1–3) (Additional file 1: Fig. S3b-c).

**Fig. 4:**
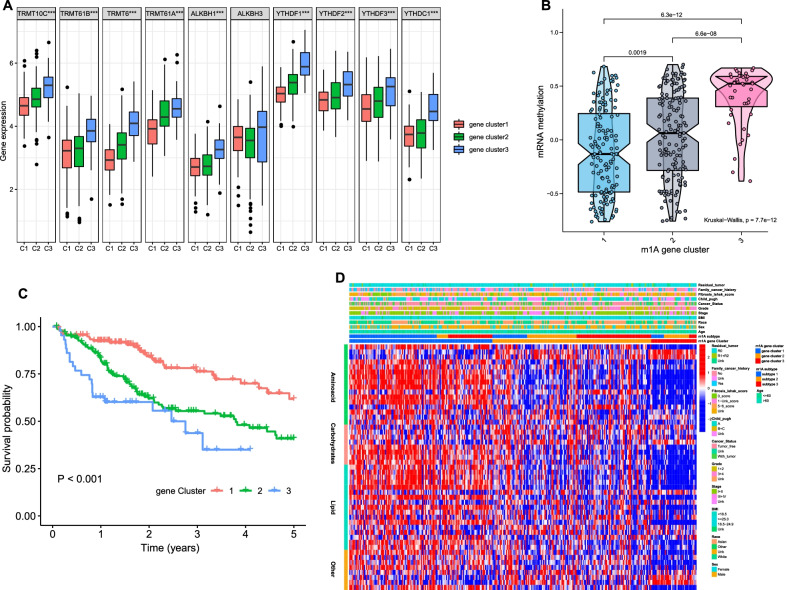
The m^1^A gene clusters in HCC. **a** Expression levels of m^1^A regulators in gene cluster 1–3. **b** Enrichment scores of mRNA methylation in gene cluster 1–3. **c** Overall survival of gene cluster 1–3 and the survival difference was evaluated by log-rank test. **d** Heatmap visualizing the gene set variation analysis enrichment scores of metabolic-related pathways in three gene clusters

The expression level of m1A regulators and mRNA methylation in the three gene clusters were estimated, and both showed significant differences (Fig. 4a, b). Survival analysis revealed that patients in gene cluster 1 had a better prognosis than patients in the other gene clusters (Fig. 4c). The metabolic characteristics of the three gene clusters were further explored, and a significant difference was observed. Gene clusters 1 and 3 had the highest and lowest enrichment scores, respectively, across the four major metabolic categories, which suggested that clusters 1 and 3 could be identified as the metabolic-high phenotype and metabolism-excluded phenotype, respectively, and cluster 2 could be identified as the metabolic-intermediate phenotype. (Fig. 4d).

**Fig. 5:**
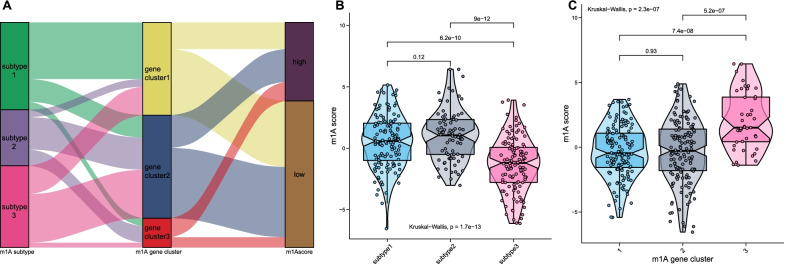
Correlation between the known signatures and m^1^Ascore. **a** Alluvial diagram visualizing the connection between m^1^Ascore, subtype1-3, and gene cluster 1–3. **b** Distribution of m^1^Ascore in subtype 1–3. **c** Distribution of m^1^Ascore in gene cluster 1–3

We further established a scoring system termed the m1Ascore. The correlation between the known classifications in this study and the m1Ascore is presented as an alluvial diagram (Fig. 5a). Patients with subtype 2 had the highest median m1Ascore compared with patients with other subtypes (Fig. 5b), indicating that a high m1Ascore could be associated with the hypoactive metabolic state. Gene cluster 3 had the highest median m1Ascore, while cluster 1 had the lowest median score (Fig. 5c). A significant correlation between the m1Ascore and metabolic pathways was directly observed (Fig. 3g).

### Risk stratification of HCC patients

**Fig. 6:**
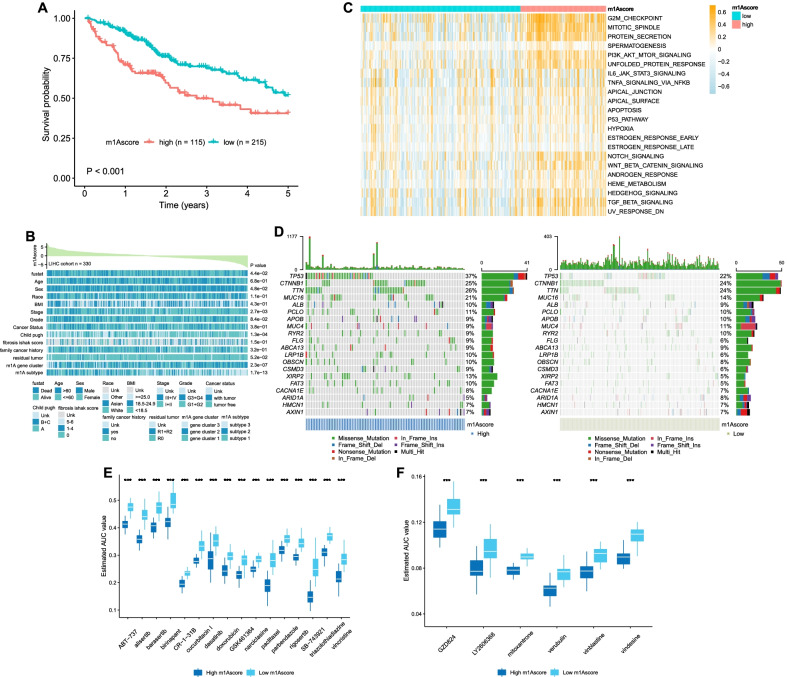
Characteristics of m^1^Ascore and drug screening. **a** Overall survival of high and low m^1^Ascore groups and the survival difference was evaluated by log-rank test. **b** Clinicopathological characteristics in high and low m^1^Ascore groups. **c** Heatmap visualizing the activation states of biological pathways in high and low m^1^Ascore groups. The pathways achieved from the HALLMARK gene sets. **d** Somatic mutation landscapes in high and low m^1^Ascore groups. **e–f** Drug response analysis of the potential compounds **e** derived from PRISM and **f** CTRP

We classified 330 patients in the TCGA cohort into high- or low-m1Ascore groups, and Kaplan–Meier (KM) curves showed that a high m1Ascore was associated with a short survival time (Fig. 6a). These results suggested the clinical value of the m1Ascore in risk stratification; thus, we named the high- and low-m1Ascore groups the high-risk group and low-risk group, respectively.

The clinicopathological information and molecular characteristics of the high-risk and low-risk groups were further assessed. Patients in the high-risk group were significantly associated with advanced stage, poor differentiation and higher Child–Pugh scores (Fig. 6b). The expression level of m1A regulators in the high-risk group was significantly higher than that in the low-risk group (Additional file 1: Fig. S4a). This result is consistent with the difference in mRNA methylation levels between the two groups (Additional file 1: Fig. S4b). A positive correlation between mRNA methylation and the m1Ascore was also observed (Additional file 1: Fig. S4c). According to GSVA, hallmarks related to cell signaling and proliferation were significantly activated in the high-risk group compared with the low-risk group, including G2M checkpoint, TGF-β signaling, Wnt-β catenin signaling and PI3K-AKT-mTOR signaling (Fig. 6c). The metabolic characteristics were investigated, and the results indicated that the low-risk group was associated with a hyperactive metabolic state, while the high-risk group was associated with a hypoactive metabolic state (Additional file 1: Fig. S4d-g). The somatic mutation landscapes are shown in Fig. 6d. TP53 showed the highest mutation frequency in the high-risk group (37%), while CTNNB1 and TTN both had the highest mutation frequencies in the low-risk group (24%). No significant differences were detected between the two groups in terms of tumor mutation burden, microsatellite instability or RNAss (Additional file 1: Fig. S4h-j).

### Identification of potential therapeutic agents

We successfully identified HCC patients with poor prognosis according to the m1Ascore; thus, this score would be valuable in the clinic to select suitable drugs for the high-risk group in the context of personalized treatment. For this purpose, 1770 compounds were retrieved for drug screening in the CTRP and PRISM databases after excluding 160 duplicated compounds. We next eliminated compounds lacking information in more than 20% of the samples. Finally, 1645 compounds were included in the subsequent analysis.

The clinical samples were classified by deciles according to the m1Ascore, and the estimated area under curve (AUC) value of each compound in each decile was obtained. Compounds with lower estimated AUC values in the top decile group were identified when compared with the bottom decile (log2FC > 0.2). Moreover, compounds that showed a negative correlation between the m1Ascore and estimated AUC values by Spearman correlation analysis were recorded (Spearman’s r < -0.40 for PRISM and CTRP). We retained 22 candidate agents after screening, including 6 agents from CTRP and 16 agents from PRISM (Fig. 6e–f). These compounds were all characterized by lower estimated AUC values and a negative correlation with the m1Ascore, representing better drug sensitivity in the high-risk HCC group. However, such features are not reliable to support the therapeutic effect of these compounds in the clinic. Hence, we subsequently performed CMap analysis, and two compounds with CMap scores < -95 were finally identified: mitoxantrone and doxorubicin. In addition, the immunotherapeutic response was also evaluated; however, no significant response difference was detected between the high-risk and low-risk groups (Additional file 1: Fig. S5a-b).

### External validation and construction of prognostic model

**Fig. 7:**
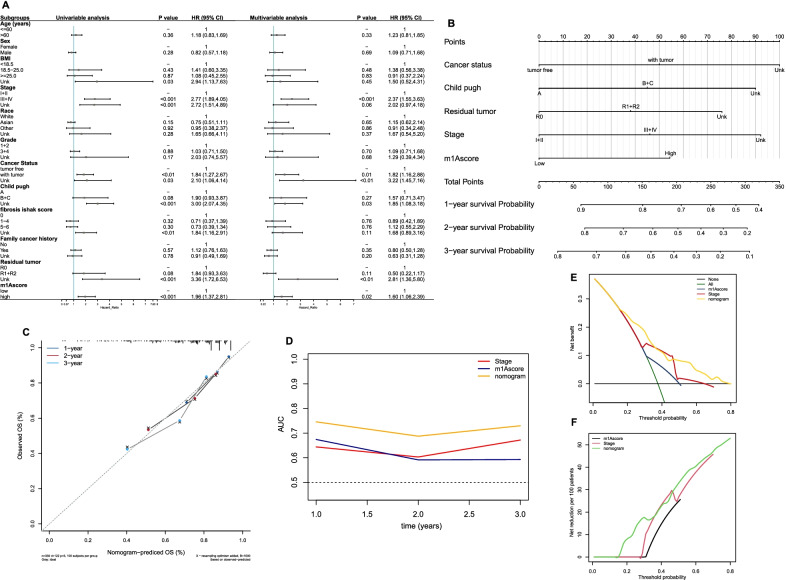
Construction of nomogram for predicting survival of HCC in TCGA cohort. **a** Forest plot showing the results of univariate and multivariate Cox analyses. **b** Nomogram. **c**Calibration plot. **d** Time-dependent receiver operating characteristic analysis and the areas under the curve at 1, 2, and 3-years. **e**, **f** Decision curve analyses. **e** Net benefit analyses. **f** Net reduction analyses

We subsequently performed multivariate Cox regression analysis, and the results demonstrated that a high m1Ascore was independently associated with poor survival (Fig. 7a). In the external validation LIRI-JP cohort, the expression levels of m1A regulators between normal and tumor tissues were analyzed, and the results confirmed the overexpression of m1A regulators in HCC patients (Additional file 1: Fig. S6a). The survival time of high-m1Ascore patients was significantly shorter than that of low-m1Ascore patients (Additional file 1: Fig. S6b). Multivariate analysis demonstrated that a high m1Ascore was an independent risk factor for survival (Additional file 1: Fig. S6c). By incorporating the other independent clinicopathological factors in the TCGA cohort, we built a nomogram for use in clinical practice (Fig. 7b). No remarkable deviation of predicted survival from actual survival was observed according to the calibration plots (Fig. 7c). tROC analysis showed that the accuracy of the nomogram in predicting survival was more satisfactory at 1, 2, and 3 years than AJCC stage or the m1Ascore alone (Fig. 7d). The AUC values of the nomogram for predicting 1-, 2-, and 3-year overall survival (OS) were 0.75, 0.69, and 0.73, respectively. In the internal validation, the adjusted C-index of the nomogram was 0.698. The decision curve analysis (DCA) results indicated that compared with the m1Ascore or AJCC stage, the utilization of the nomogram in the clinic had greater net benefits and net reduction (Fig. 7e–f). These results suggested that the nomogram performed well.

### Pan-cancer analysis

**Fig. 8:**
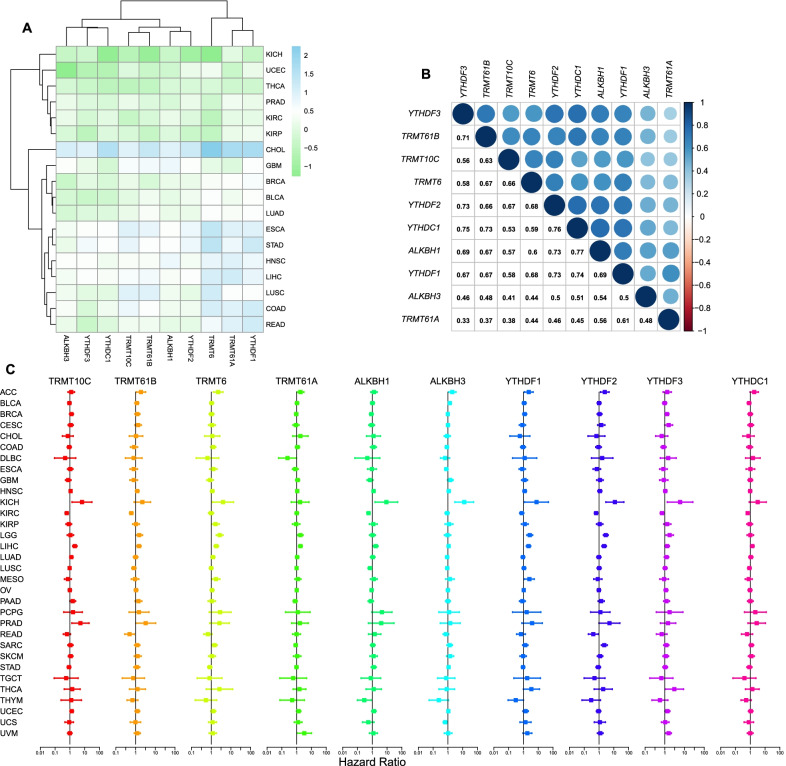
Pan-cancer analyses. **a** Heatmap showing the expression levels of m^1^A regulators in tumor tissues compared with normal tissues in 18 cancer types. **b **Expression correlation of m^1^A regulators in 33 cancer types. **c **Forest plots showing the results of univariate Cox analysis in 33 cancer types

**Fig. 9:**
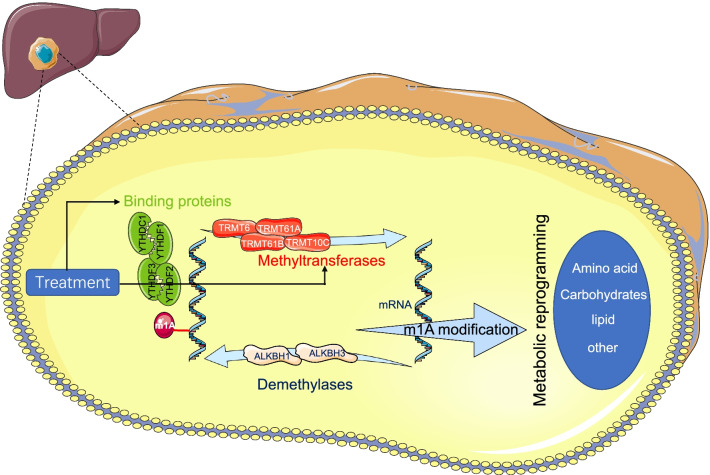
A schematic of m1A regulators in liver cancer cells. A methyl residue is added to mRNA by methyltransferases, removed by demethylases, and recognized by binding proteins. The modification of m1A methylation finally results in metabolic reprogramming in hepatocellular carcinoma. Images used in the current schematic are freely taken from Servier Medical Art (https://smart.servier.com/)

We further chosen data on 18 cancer types that had more than 5 normal samples from the TCGA database to comprehensively investigate the expression patterns of the m1A regulators [21]. The expression distributions of the m1A regulators across all cancers are shown in Additional file 1: Fig. S7a, and the expression landscapes are shown in Fig. 8a. The expression level of each regulator was significantly upregulated in cholangiocarcinoma (CHOL) compared with the control, while downregulation was observed in kidney chromophobe (KICH) and thyroid carcinoma (THCA). In kidney renal clear cell carcinoma (KIRC), compared with normal tissues, we noticed that the expression levels of TRMT10C and TRMT6 were significantly higher, while those of ALKBH1, ALKBH3 and YTHDF1 were significantly lower (Additional file 1: Fig. S7b). The expression correlations among m1A regulators across cancers are shown in Fig. 8b, in which ALKBH1 and YTHDC1 had the highest association (r = 0.77, P < 0.01). These results indicated a prevalent m1A regulator expression difference between normal and tumor tissues, and the expression level of each regulator showed remarkable intratumor heterogeneity. Univariate Cox regression analysis was performed on 33 cancer types to explore the impact of m1A regulators on survival, and the results showed that the prognostic value of each regulator varied across cancer types (Fig. 8c). For example, overexpression of YTHDF2 and ALKBH1 is associated with poor survival in KICH and HCC but with better survival in KIRC. Finally, to better understanding the influence of m1A regulators in cancer biology, we provided a schematic to summarize the current knowledge of the roles of m1A regulators in metabolic reprogramming of hepatocellular carcinoma and pointed the potential therapeutic targets (Fig. 9).

## Discussion

m1A, which has been reported to play a critical role in a variety of functional activities, has drawn increasing interest in recent years. In RNA metabolism, it was found that the recruitment of the m1A reader YTHDF2 induces the degradation of m1A-modified RNAs [22]. In translation processes, it was reported that m1A modification was enriched in the region near the start codon, and these sites tended to form secondary structures with higher translation efficiency [3]. However, the impact of m1A in cancer biology has been poorly elucidated to date.

Here, we first reported three distinct m1A modification patterns in HCC patients and found that each pattern had distinct metabolic characteristics. Subtype 1 displayed the highest metabolic activity across the four major metabolic categories, characterized by a metabolism-high phenotype. Subtype 2 displayed the lowest metabolic activity, characterized by a metabolism-excluded phenotype. Subtype 3 displayed moderate metabolic activity, characterized by a metabolism-intermediate phenotype. Survival analysis showed that patients with subtype 2 had the worst prognosis compared with patients with the other subtypes. Yang C et al. reported three HCC subclasses (subclasses 1–3) with distinct metabolic characteristics based on the expression profiles of 2752 metabolism-related genes [23]. Subclass 3, which displayed a hypoactive metabolic state, had a poorer prognosis than subclass 1. Shen et al. reported three clusters in HCC patients with distinct metabolic characteristics using the expression data of m6A regulators [24]. Cluster 3 showed the worst OS and the lowest metabolic activity. The findings of these studies are consistent with our results. We also observed that among seven major processes of cancer, hallmarks related to metabolism, including fatty acid metabolism, xenobiotic metabolism and bile acid metabolism, were significantly downregulated in subtype 2 compared with the other subtypes. Combined with the metabolic characteristics of each subtype, these results demonstrated the hypoactive metabolism of subtype 2, favoring the credibility of our classification of metabolic phenotypes for different m1A modification patterns. We further elucidated the association between m1A modification patterns and metabolic characteristics by analyzing the m1A subtype-related DEGs. The three genomic patterns were also found to be related to distinct metabolic states and prognoses, which again confirmed the metabolic heterogeneity of each m1A modification pattern.

We next developed a scoring system, the m1Ascore, and found that subtype 2 and gene cluster 3 had the highest median scores among the three subtypes and gene clusters. As expected, patients belonging to the high-m1Ascore group had highly expressed m1A regulators with a hypoactive metabolic state. These results suggested that the m1Ascore could be used as a tool to estimate the m1A modification pattern in individual patients, further reflecting the metabolic state. Further analysis confirmed that the m1Ascore was an independent risk factor for HCC patient prognosis, and a nomogram incorporating the m1Ascore and other clinicopathological factors was successfully constructed and showed good discriminatory capacity and accuracy. These results showed that m1A regulators contribute to the prognosis of patients with HCC. The roles of m1A regulators in HCC also had been investigated in previous studies. The expression levels of readers, including YTHDF1, YTHDF2, and YTHDF3, are reported to upregulate in HCC tissues compared with adjacent tissues, and a positive correlation was detected between immune cell infiltration and the expression levels of YTHDF1, YTHDF2, and YTHDF3, revealing the functional roles of m1A regulators in modulating the infiltration of immune cells in HCC tissues [25]. As for erasers, consistent with our results, Wang et al. reported an elevated ALKBH3 expression in HCC compared with adjuvant non-tumorous tissues, and found that patients with high ALKBH3 expression level displayed poor prognosis and the knockdown of ALKBH3 inhibits tumor cells proliferation, indicating the functional role of m1A modification in promoting cell cycle [26]. A recent study reported that the expression levels of writers TRMT6 and TRMT61A in HCC tissues were significantly increased and showed that the increased TRMT6 and TRMT61A expression levels are negatively associated with patients’ prognosis, and found that TRMT6/TRMT61A-mediated m1A methylation can initiate liver tumorigenesis by regulating lipid metabolism [27]. The modification effect of ALKBH3 on m1A methylation is opposite to that of TRMT6 and TRMT61A, however, as revealed by the above studies and our results, a poor prognosis was consistently observed in HCC patients with an elevated TRMT6/ TRMT61A or ALKBH3 expression level, suggesting the crucial role of m1A methylation dysfunction in HCC. It has been reported that m1A modulates the PI3K/AKT/mTOR pathway in gastrointestinal cancers [28], and the dysregulation of this pathway has been identified as a key factor contributing to poor survival in several cancers [29–31]. Thus, the mechanism underlying the impact of m1A on survival is probably associated with the dysregulation of the PI3K/AKT/mTOR pathway. Moreover, the PI3K/AKT/mTOR pathway is known as an important process in regulating glucose intake [32] and is deemed a crosstalk center between epigenetics and metabolic heterogeneity for the ability to drive the Warburg effect [33]. Further research on the PI3K/AKT/mTOR pathway is needed to enhance our understanding of the metabolic heterogeneity between each m1A modification pattern. In addition, consistent with the results reported by Shi et al. that CNV events commonly occurred in m1A regulators in HCC [34], our study found a prevalence of CNVs in all m1A regulators, indicating that the alternations to m1A regulators in HCC are prevalent. It had been reported that recessive mutations in TRMT10C affect aerobic respiration processing and result in lactic acidosis [35]. However, very few studies have focused on the effects of the genetic alternations of m1A regulators on HCC biology to date. TP53 mutation, which has been demonstrated as an important tumorigenesis procedure [36] and to enhance aerobic glycolysis of tumor cells [37], was found to positively correlate with the genetic changes of m1A regulators in HCC, and mutation frequency of TP53 in low- and high-m1Ascore groups were also found significantly different in our study (22% vs. 37%, P = 0.003). These results promoted us that the genetic variants of m1A regulators might cooperate with the mutations of cancer-causing genes in the oncogenesis and metabolic reprogramming of HCC.

Identifying promising treatment strategies based on molecular characteristics to maximize the therapeutic effect is also one of the main purposes of this study. We subsequently identified two potential agents for the treatment of high-risk HCC patients, mitoxantrone and doxorubicin. Mitoxantrone is an antineoplastic drug that is widely used in treating acute myeloid leukemia (AML) [38] and breast cancer [39]. The efficacy of mitoxantrone for HCC patients has already been investigated [40, 41], and the results suggested that patients with smaller tumor masses and good liver reserves may benefit from a therapeutic regimen consisting of mitoxantrone, 5-fluorouracil and cisplatin. Our study provided new insights to identify patients suitable for treatment with mitoxantrone. Doxorubicin is one of the first-line chemotherapeutic agents used in the management of hematological tumors [42] and has also been approved for the treatment of HCC patients [43]. Cardiotoxicity is a common side effect in the application of doxorubicin [44]. It has been reported that 48% of patients using 700 mg/m^2^ doxorubicin will develop heart failure [45]. Our study indicated that the clinical use of doxorubicin in patients with a high m1Ascore might be more reasonable compared with that in patients with a low m1Ascore. This result is helpful for reducing the risk of cardiotoxicity in patients and avoiding ineffective overtreatment.

There are some limitations in the current study. First, we estimated the metabolic characteristics of each m1A modification pattern using the GSVA score of metabolic pathways rather than directly analyzing the metabolite profiling or expression patterns of metabolic genes. Some metabolic pathways related to fatty acid metabolism, which shared common metabolic-related genes, might have similar scores. Thus, we have to acknowledge that there might be discrepancies to a certain extent between the estimated metabolic state and actual metabolic state of each pattern. Second, the impacts of each m1A regulator on key metabolic enzymes were not discussed here, and the molecular mechanism underlying the correlation between m1A modification and metabolic heterogeneity remains unknown. Finally, the results of the current study are all based on bioinformatics analyses. The lack of experimental verification might undermine the persuasiveness of our conclusion; hence, further in vivo studies are needed to promote the application of our findings in clinical practice.

## Conclusion

Our findings highlighted the role of m1A methylation modification in the crosstalk between epigenetics and metabolic heterogeneity in cancer. The estimation of m1A modification in HCC patients will promote our understanding of metabolic characteristics and be beneficial for survival stratification, further guiding personalized clinical treatment.

## Supplementary Information

**Additional file 1: Table S1. **Clinical Characteristics of the HCC patients in TCGA and ICGC; **Figure S1.** Expression levels of m1A regulators in HCC and normal tissues; **Figure S2.** Unsupervised clustering of m1A regulators and metabolic characteristics in each cluster; **Figure S3.** m1A subtype-related overlap genes and unsupervised clustering of the overlap genes; **Figure S4.** Characteristics in high and low m1Ascore groups; **Figure S5.** Difference in immunotherapy response between high- and low-m1Ascore groups; **Figure S6.** External validation of m1Ascore in ICGC-JP cohort; **Figure S7.** Expression levels of m1A regulators in TCGA cohort across pan-cancer analysis.

## Data Availability

The datasets were downloaded from the The Cancer Genome Atlas (TCGA) database (https://www.cancer.gov/) and International Cancer Genome Consortium (ICGC) database (https://dcc.icgc.org/).

## Abbreviations

HCC: Hepatocellular carcinoma (HCC);
m1A: N1-methyladenosine
TCGA: The Cancer Genome Atlas
ICGC: International Cancer Genome Consortium
CNV: Copy number variation
GSVA: Gene set variation analysis
DEGs: Different expression genes
KM: Kaplan–Meier
AUC: Area under curve
DCA: Decision curve analysis
