# Supplementary material for: m1A methylation modification patterns and metabolic characteristics in hepatocellular carcinoma
Source: BMC Gastroenterol. 2022 Mar 3;22:93. doi: 10.1186/s12876-022-02160-w (PMC8896097; doi:10.1186/s12876-022-02160-w)
Supplement: Supplementary file 1 — Additional file 1: Table S1. Clinical Characteristics of the HCC patients in TCGA and ICGC; Figure S1. Expression levels of m1A regulators in HCC and normal tissues; Figure S2. Unsupervised clustering of m1A regulators and metabolic characteristics in each cluster; Figure S3. m1A subtype-related overlap genes and unsupervised clustering of the overlap genes; Figure S4. Characteristics in high and low m1Ascore groups; Figure S5. Difference in immunotherapy response between high- and low-m1Ascore groups; Figure S6. External validation of m1Ascore in ICGC-JP cohort; Figure S7. Expression levels of m1A regulators in TCGA cohort across pan-cancer analysis. [file 12876_2022_2160_MOESM1_ESM.pdf]

m1A methylation modification patterns and metabolic characteristics in hepatocellular carcinoma
Supplementary information

Chengcheng Tong#, Wei Wang#*, Chiyi He*
Department of Gastroenterology, Yijishan Hospital, Wannan Medical College, Wuhu, Anhui Province, China

*** Correspondence**
Chiyi He: hechiyi11@wnmc.edu.cn
Wei Wang: wwwy@wnmc.edu.cn

#These authors have contributed equally to this work

Supplementary materials and methods

Data collection

Two RNA sequencing (RNA-seq) datasets were used in the current study: The Cancer Genome Atlas (TCGA, liver hepatocellular carcinoma (LIHC), https://www.cancer.gov/) and International Cancer Genome Consortium (ICGC) (LIRI-JP, www.icgc.org). HCC patients with RNA-seq data (fragments per kilobase per million reads, FPKM) and corresponding clinical information were enrolled in March 2021. Patients diagnosed with primary solid HCC with age ≥ 18 years were included, and patients with a history of preoperative adjuvant therapy, those lacking survival time information or those with a survival time of less than 30 days were excluded. In case of different samples derived from one patient were also excluded. Finally, we enrolled 330 patients from the TCGA cohort and 194 patients from the LIRI-JP cohort. The clinicopathological information of all patients is listed in Table S1. We also obtained somatic mutation data (MuTect 2) and copy number variation (CNV) data from the TCGA database. The mutation landscapes were presented as waterfall plots using the "maftools" package, and the CNV landscape of the m1A regulators was plotted by the "RCircos" package. In addition, the RNA-seq data and corresponding survival information of 33 types of cancer were downloaded from the TCGA database for pan-cancer analysis. FPKM values were transformed into transcripts per kilobase million (TPM) values.

Unsupervised clustering of m1A regulators and identification of differentially expressed genes (DEGs)

Ten genes that were reported as m1A modification regulators were retrieved, including 4 methyltransferases (TRMT10C, TRMT61B, TRMT6 and TRMT61A), which are also called "writers", 2 demethylases (ALKBH1 and ALKBH3), which are also called "erasers", and 4 binding proteins (YTHDF1, YTHDF2, YTHDF3 and YTHDC1), which are also called "readers". Unsupervised cluster analysis was performed in the TCGA cohort using the "ConsensusClusterPlus" package to identify distinct m1A modification patterns based on the expression of the 10 m1A regulators. The "limma" package was used to investigate the DEGs between distinct m1A modification patterns. The DEGs were determined by an adjusted value of $P < 0.05$ and $|\log_2 \text{ fold change (FC)}| < 1$.

Gene set variation analysis (GSVA)

In the HALLMARK gene sets, 50 hallmarks, which represent 50 well-defined biological activities, were classified into seven major processes related to cancer [1]. We used the "GSVA" package to detect the enrichment difference in each hallmark between different m1A modification patterns and identified processes associated with each pattern. Shen et al reported 49 metabolism-associated pathways and divided these pathways into four major metabolic categories, including carbohydrate, lipid, amino acid and other metabolic categories [2]. We obtained these pathways from the Kyoto Encyclopedia of Genes and Genomes (KEGG) and BIOCARTA gene sets and calculated an enrichment score corresponding to each metabolism-associated pathway for each sample by GSVA. In addition, GSVA was also performed based on the mRNA methylation gene signatures in GOBP to investigate the mRNA methylation level. All gene sets were downloaded from the Molecular Signatures Database (MSigDB) (https://www.gsea-msigdb.org/gsea/msigdb/index.jsp), and a statistically significant difference was defined as adjusted $P < 0.05$.

Generation of an m1A scoring system

We generated a scoring system for the clinical estimation of the m1A modification pattern and metabolic characteristics of individual patients–the m1Ascore. The procedures were as follows: first, overlapping genes were extracted from the DEGs, and unsupervised cluster analysis was performed based on the overlapping genes to group patients into different clusters. Next, we defined the overlapping genes that had positive and negative correlations with the cluster signature as gene signatures A and B and used the Boruta algorithm to reduce the dimensions of gene signatures A and B. Finally, principal component analysis (PCA) was conducted to generate the scoring system. Principal component 1 was selected, and the m1Ascore was defined as[3]:

$$m1A\ score = \sum PC1_{A} - \sum PC1_{B}$$

Estimation of drug sensitivities

The Cancer Therapeutic Response Portal (CTRP)[4] and PRISM [5]datasets are two large pharmacogenomic databases that provide drug sensitivity data for thousands of compounds in terms of the area under the dose-response curve (AUC) values. Patients who showed lower estimated AUC values had higher rates of clinical benefit from the given compound[6]. We collected compounds from the CTRP and PRISM datasets (cell lines derived from hematopoietic and lymphoid tissues were excluded) and applied the ridge-regression model in the "pRRophetic" package to obtain the estimated AUC value of each compound in each sample for drug screening[7]. CMap analysis was applied to generate a connective score which ranging from -100 to 100 for each compound, and compounds with scores < -95 indicated that patients might benefit from this intervention[8]. We applied the Tumor Immune Dysfunction and Exclusion (TIDE) algorithm[9] and subclass mapping[10] to predict the patient response to immune checkpoint inhibitors. For subclass mapping, the data of 47 patients with melanoma were obtained [11].

Statistical analysis

R 4.0.1 was applied to carry out all statistical tests. The Wilcoxon rank-sum test and Kruskal-Wallis test were used for comparisons of continuous variables between two groups and more than two groups, respectively. Comparisons of categorical variables were performed by the chi-square test or Fisher's exact test. Correlations between two continuous variables that were normally distributed were evaluated by Pearson's r correlation, and variables that were nonnormally distributed were evaluated by Spearman's rank order correlation. The Kaplan-Meier (KM) method was used to perform survival analysis, and the log-rank test was used to detect significant differences in survival. Cox proportional hazards regression analysis was used to identify parameters with prognostic value, and parameters identified as independent factors were used to establish a nomogram. Time-dependent receiver operating characteristic (tROC) analysis and calculation of the areas under the curve at different time points [AUC(t)] were performed via the "survival ROC" package. The performance of the nomogram was also assessed by the calibration curves and decision curve analysis (DCA). The bootstrapping method (1000 resamples) was performed for internal validation. All tests were two-sided, and a P value < 0.05 was considered statistically significant.

Supplementary references

1. Liberzon A, Birger C, Thorvaldsdóttir H, Ghandi M, Mesirov JP, Tamayo P. The Molecular Signatures Database (MSigDB) hallmark gene set collection. *Cell Syst*. 2015;1(6):417-425. doi:10.1016/j.cels.2015.12.004

2. Shen X, Hu B, Xu J, et al. The m6A methylation landscape stratifies hepatocellular carcinoma into 3 subtypes with distinct metabolic characteristics. *Cancer Biol Med*. 2020;17(4):937-952. doi:10.20892/j.issn.2095-3941.2020.0402

3. Zhang X, Shi M, Chen T, Zhang B. Characterization of the Immune Cell Infiltration Landscape in Head and Neck Squamous Cell Carcinoma to Aid Immunotherapy. *Mol Ther Nucleic Acids*. 2020;22:298-309. doi:10.1016/j.omtn.2020.08.030

4. Garnett MJ, Edelman EJ, Heidorn SJ, et al. Systematic identification of genomic markers of drug sensitivity in cancer cells. *Nature*. 2012;483(7391):570-575. doi:10.1038/nature11005

5. Yu C, Mannan AM, Yvone GM, et al. High-throughput identification of genotype-specific cancer vulnerabilities in mixtures of barcoded tumor cell lines. *Nat Biotechnol*. 2016;34(4):419-423. doi:10.1038/nbt.3460

6. Yang C, Huang X, Li Y, Chen J, Lv Y, Dai S. Prognosis and personalized treatment prediction in TP53-mutant hepatocellular carcinoma: an in silico strategy towards precision oncology. *Brief Bioinform*. Published online August 13, 2020. doi:10.1093/bib/bbaa164

7. Shen R, Li P, Li B, Zhang B, Feng L, Cheng S. Identification of Distinct Immune Subtypes in Colorectal Cancer Based on the Stromal Compartment. *Front Oncol*. 2019;9:1497. doi:10.3389/fonc.2019.01497

8. Li H, Shi X, Jiang H, et al. CMap analysis identifies Atractyloside as a potential drug candidate for type 2 diabetes based on integration of metabolomics and transcriptomics. *J Cell Mol Med*. 2020;24(13):7417-7426. doi:10.1111/jcmm.15357

9. Jiang P, Gu S, Pan D, et al. Signatures of T cell dysfunction and exclusion predict cancer immunotherapy response. *Nat Med*. 2018;24(10):1550-1558. doi:10.1038/s41591-018-0136-1

10. Hoshida Y, Brunet J-P, Tamayo P, Golub TR, Mesirov JP. Subclass mapping: identifying common subtypes in independent disease data sets. *PloS One*. 2007;2(11):e1195. doi:10.1371/journal.pone.0001195

11. Hugo W, Zaretsky JM, Sun L, et al. Genomic and Transcriptomic Features of Response to Anti-PD-1 Therapy in Metastatic Melanoma. *Cell*. 2016;165(1):35-44. doi:10.1016/j.cell.2016.02.065

Table S1: Clinical Characteristics of the HCC patients in TCGA and ICGC.

(A) Clinical characteristics of LIHC cohort (n=330).

Characteristics	Patients 330 (100%)	Characteristics	Patients 330 (100%)
**Age**		**Family cancer history**	
<= 60	160(48.5%)	no	187 (56.7%)
>60	170(51.5%)	yes	101 (30.6%)
**Sex**		Unknown	42 (12.7%)
Female	105 (31.8%)	**Residual tumor**	
Male	225 (68.2%)	R0	294 (89.1%)
**Race**		R1+R2	14 (4.2%)
White	157 (47.6%)	Unknown	22 (6.7%)
Asian	147 (44.5%)	**m1A score**	
other	16 (4.9%)	low	215 (65.2%)
Unknown	10 (3.0%)	high	115 (34.8%)
**BMI**		**Cancer status**	
<18.5	18 (5.5%)	tumor free	206 (62.4%)
18.5-25	140 (42.4%)	with tumor	103 (31.2%)
>=25	148 (44.8%)	Unknown	21 (6.4%)
Unknown	24 (7.3%)	**Child pugh**	
**Stage**		A	198 (60.0%)
1+2	227 (68.8%)	B+C	20 (6.1%)
3+4	81 (24.5%)	Unknown	112 (33.9%)
Unknown	22 (6.7%)	**Fibrosis ishak score**	
**Grade**		0	67 (20.3%)
1+2	205 (62.1%)	1-4	54 (16.4%)
3+4	120 (36.4%)	5-6	70 (21.2%)
Unknown	5 (1.5%)	Unknown	139 (42.1%)

(B) Clinical characteristics of LIRI-JP cohort (n=194).

Characteristics	Patients 194 (100%)	Characteristics	Patients 194 (100%)
**Age**		**Cancer history**	
<= 60	43 (22.2%)	no	126 (65.0%)
> 60	151 (77.8%)	yes	68 (35.0%)
**Sex**		**Prior maligancy**	
Female	50 (25.8%)	no	171 (88.1%)
Male	144 (74.2%)	yes	23 (11.9%)
**Stage (LCSGJ)**		**m1A score**	
1+2	123 (63.4%)	low	166 (85.6%)
3+4	71 (36.6%)	high	28 (14.4%)
**Treatment**			
no	140 (72.2%)		
yes	54 (27.8%)		

LCSGJ：Liver Cancer Study Group of Japan

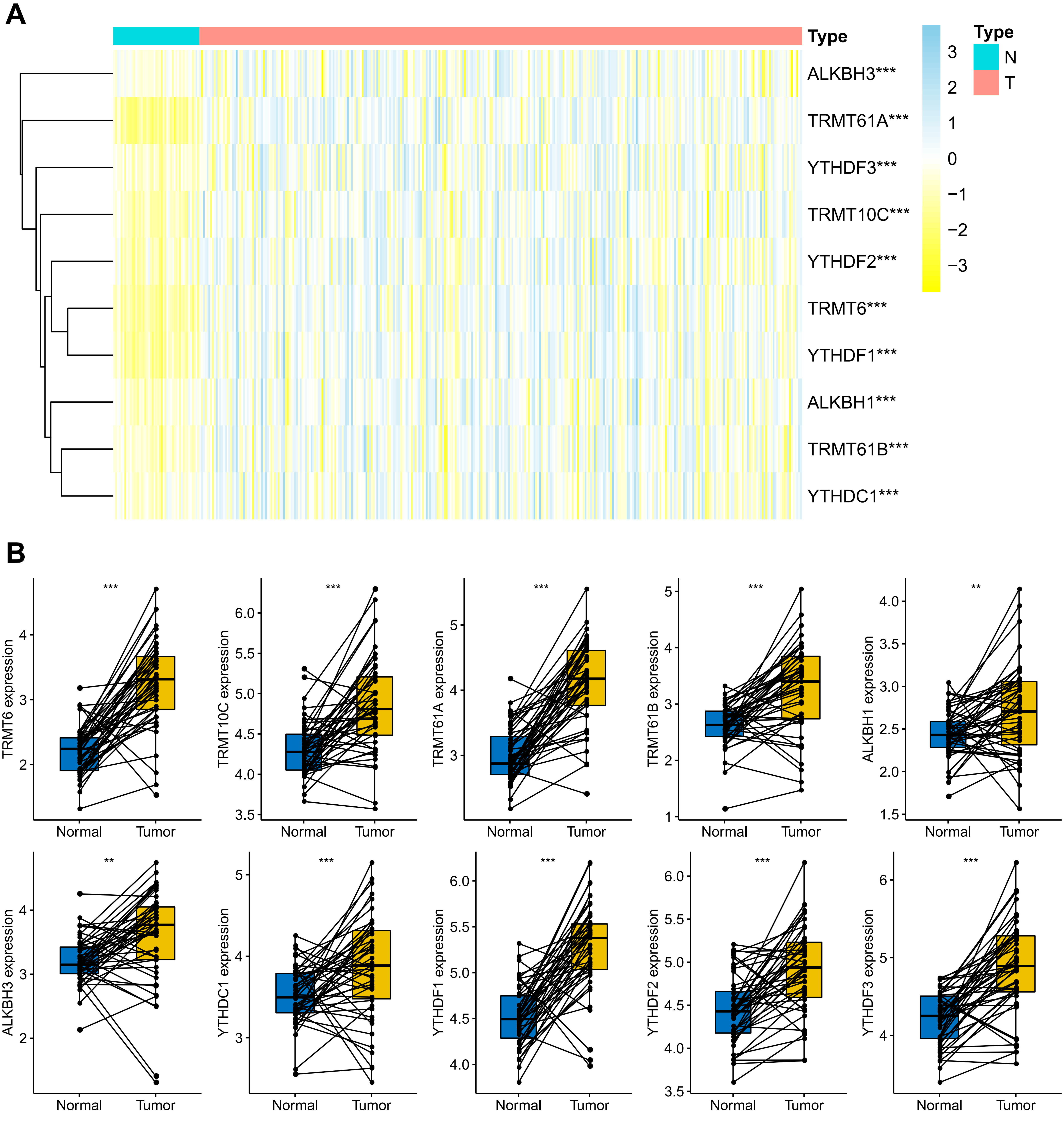

Figure S1. Expression levels of m1A regulators in HCC and normal tissues. (A) Heatmap visualizing the expression levels of m1A regulators in HCC and normal tissues. (B) Expression levels of m1A regulators in pairs HCC and adjacent normal tissues.

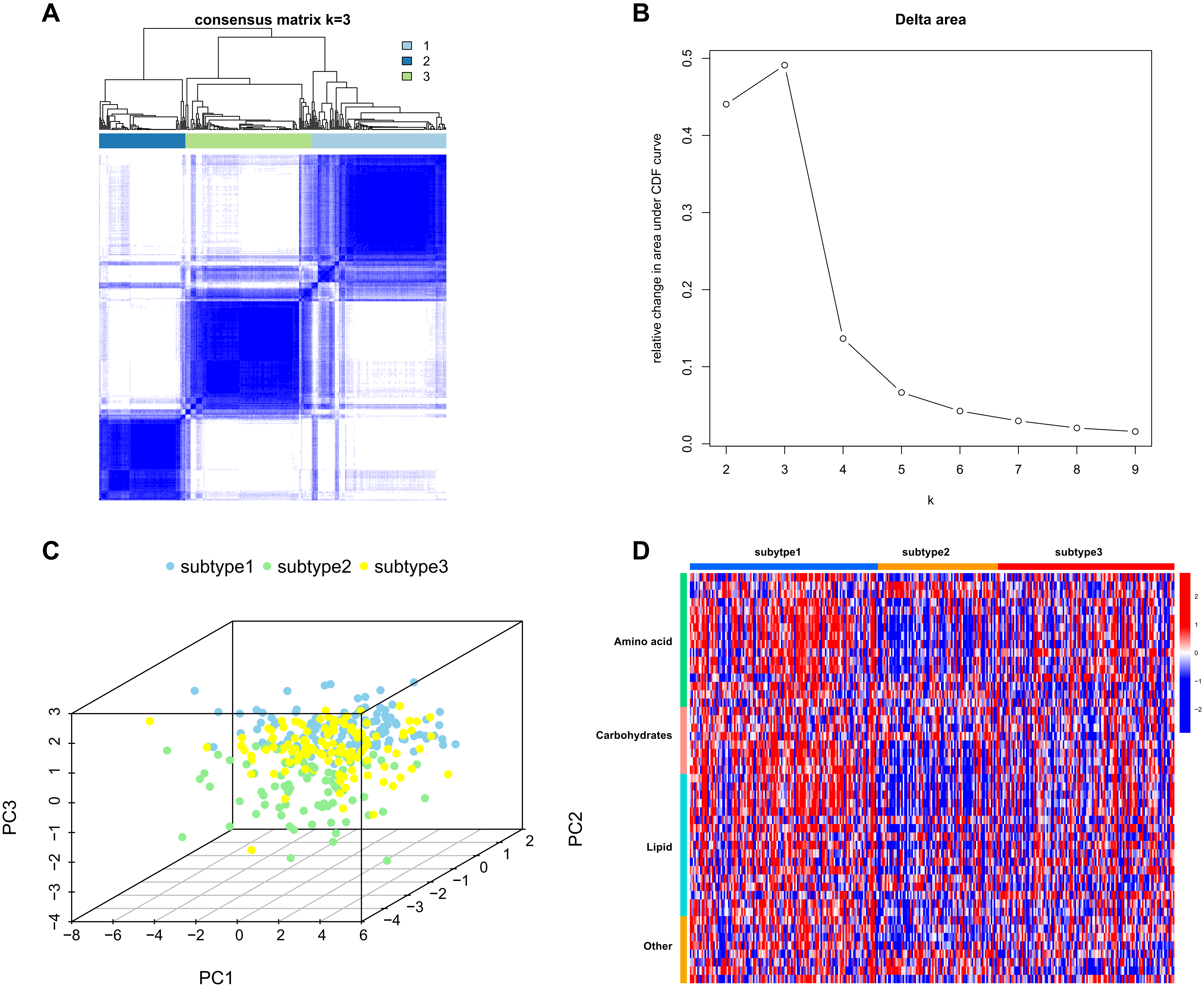

Figure S2. Unsupervised clustering of m1A regulators and metabolic characteristics in each cluster. (A) Consensus matrices for k = 3. (B) Delta area curve. (C) Principal component analysis of m1A regulators identified three m1A modification patterns. (D) Heatmap visualizing the gene set variation analysis enrichment scores of metabolic-related pathways in subtype 1-3.

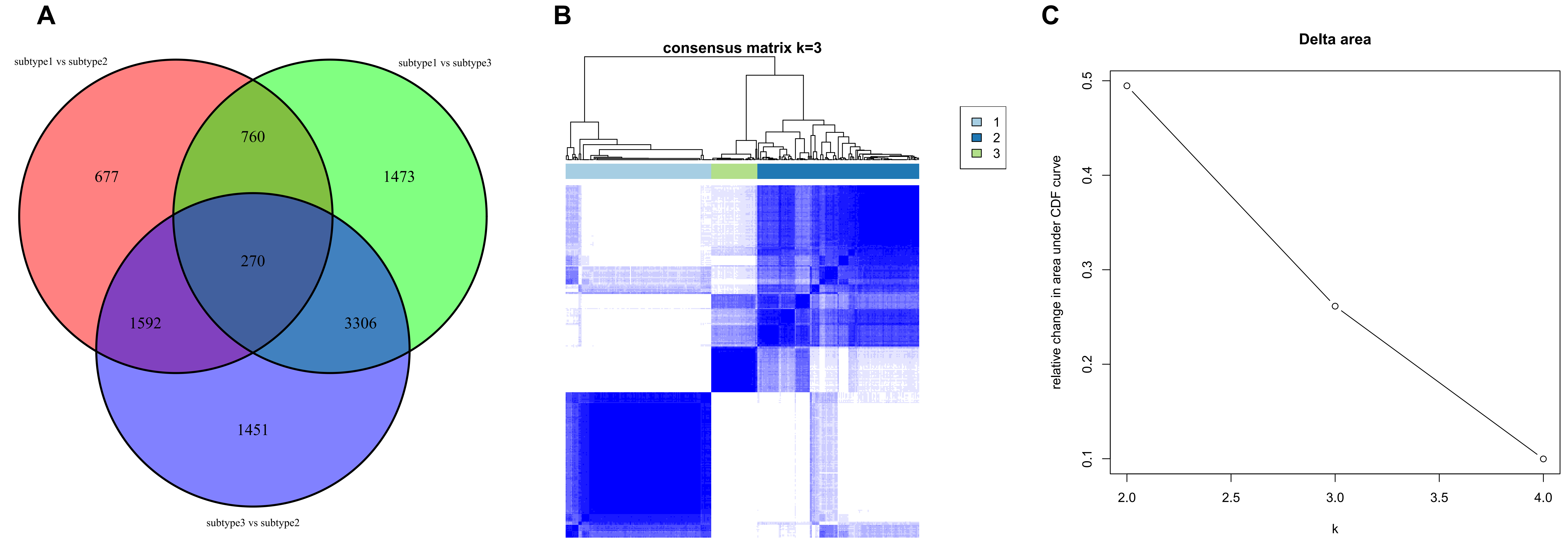

Figure S3. m1A subtype-related overlap genes and unsupervised clustering of the overlap genes.
(A) Venn diagram visualizing 270 m1A subtype-related overlap genes. (B) Consensus matrices for k = 3. (C) The Delta area curve.

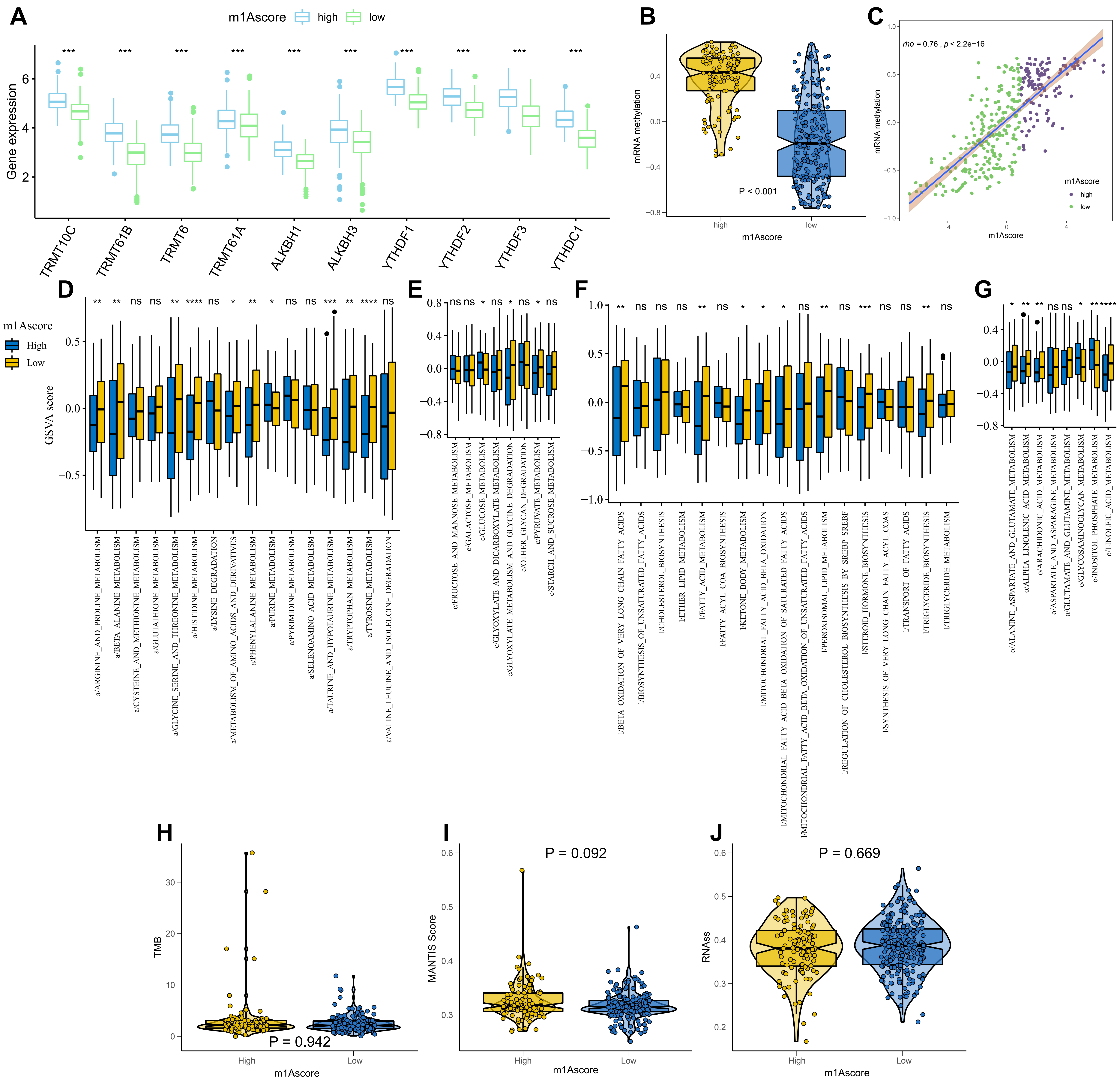

Figure S4. Characteristics in high and low m1Ascore groups. (A) Expression levels of m1A regulators in high- and low-m1Ascore groups. (B) Enrichment scores of mRNA methylation in high- and low-m1Ascore groups. (C) Correlation between m1Ascore and enrichment scores of mRNA methylation. (D-G) Gene set variation analysis enrichment scores of metabolic-related pathways in subtype 1-3. (D) Amino acid metabolism. (E) Carbohydrate metabolism. (F) Fatty acid metabolism. (G) Others metabolism. (H) Difference in TMB between high- and low-m1Ascore groups. (I) Difference in MANTIS score between high- and low-m1Ascore groups. (J) Difference in cancer stemness RNAss score between high- and low-m1Ascore groups.

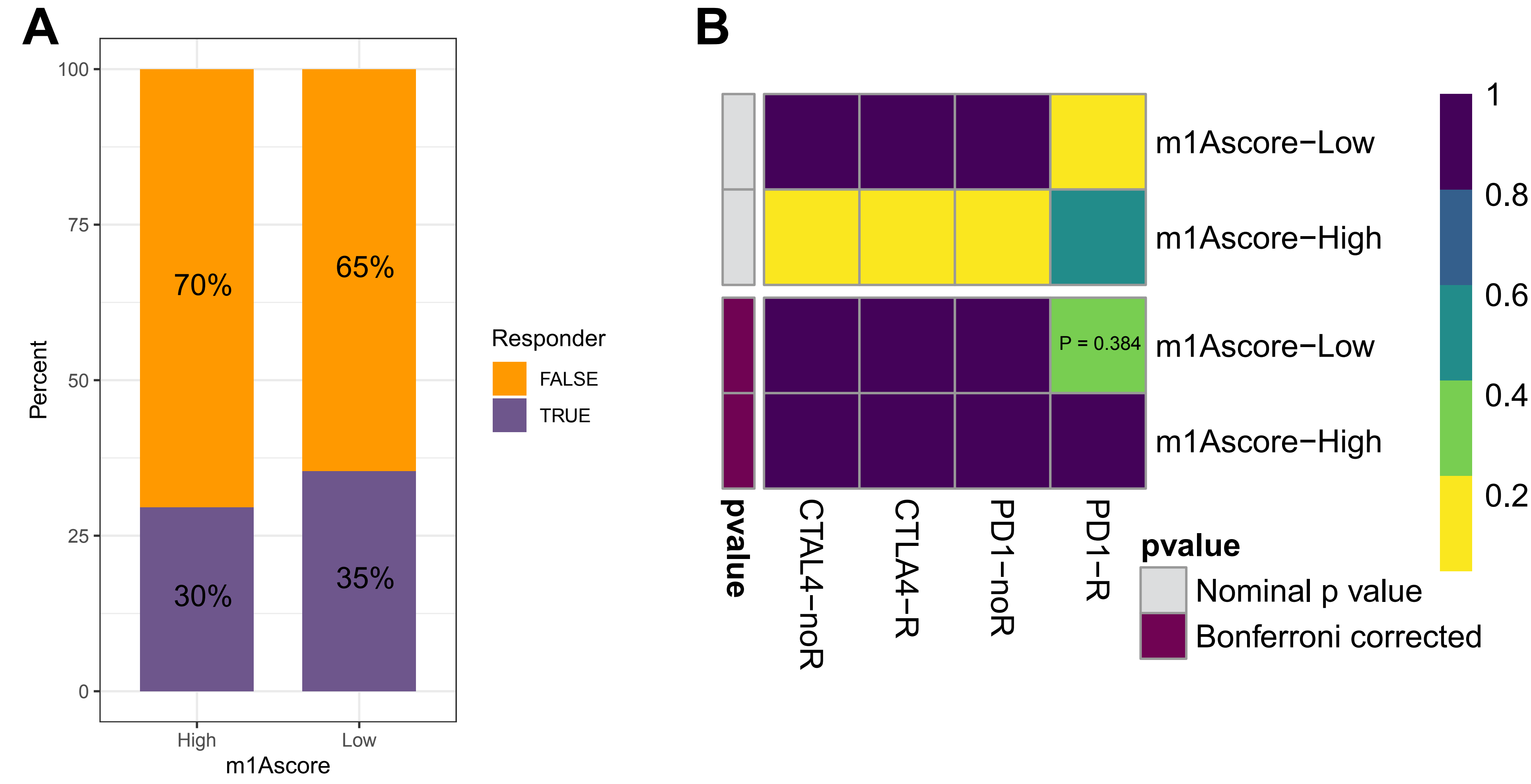

Figure S5. Difference in immunotherapy response between high- and low-m1Ascore groups.
(A) Barplots visualizing the proportion of patients responded to immunotherapy in high- and low-m1Ascore groups.
(B) Subclassing mapping showing no significant difference in the proportion of patients responded to immunotherapy between high- and low-m1Ascore groups.

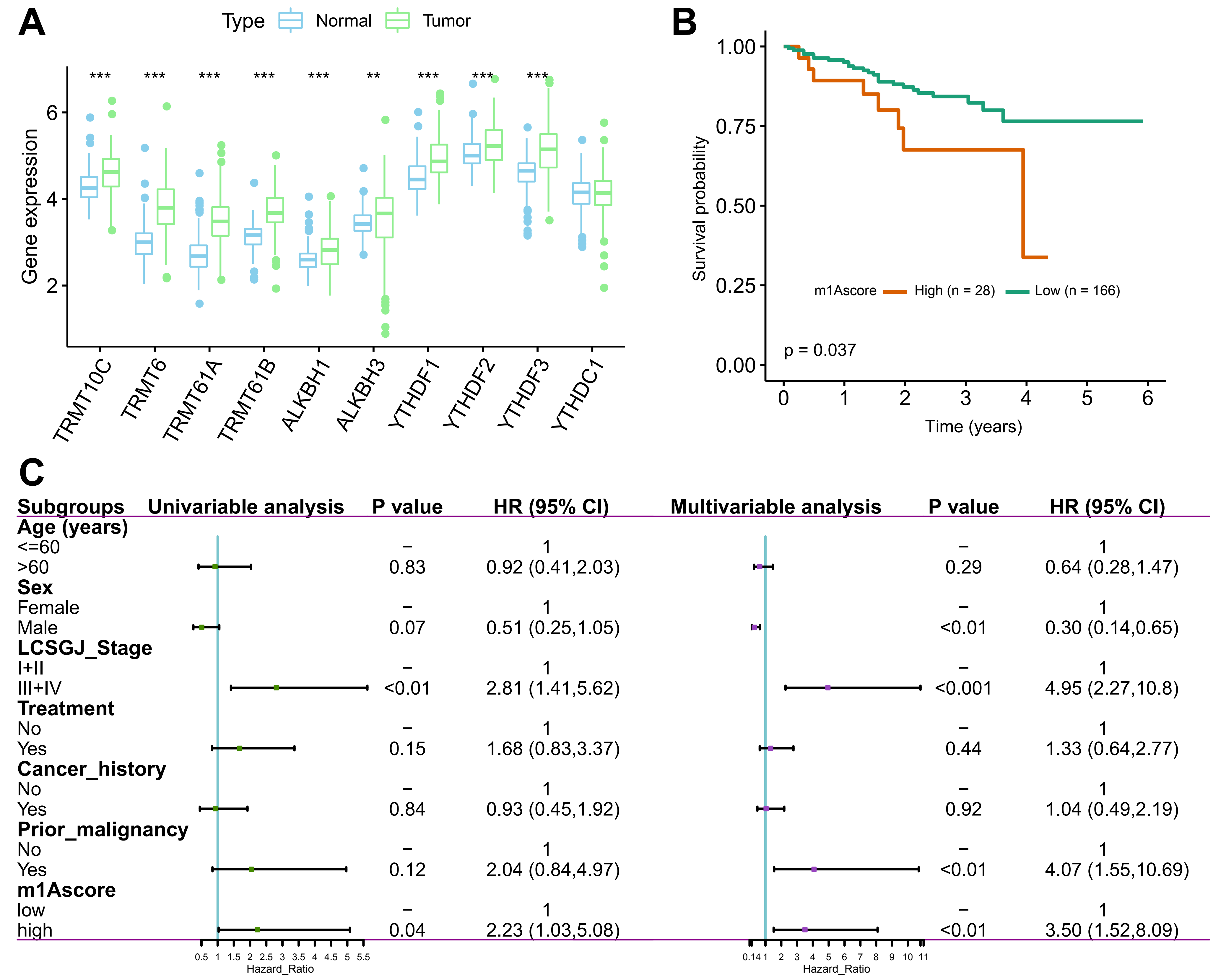

Figure S6. External validation of m1Ascore in ICGC-JP cohort. (A) Expression levels of m1A regulators in hepatocellular carcinoma and normal tissues. (B) Overall survival of high- and low-m1Ascore groups and the survival difference was evaluated by log-rank test. (C) Forest plot showing the results of univariate and multivariate Cox analyses.

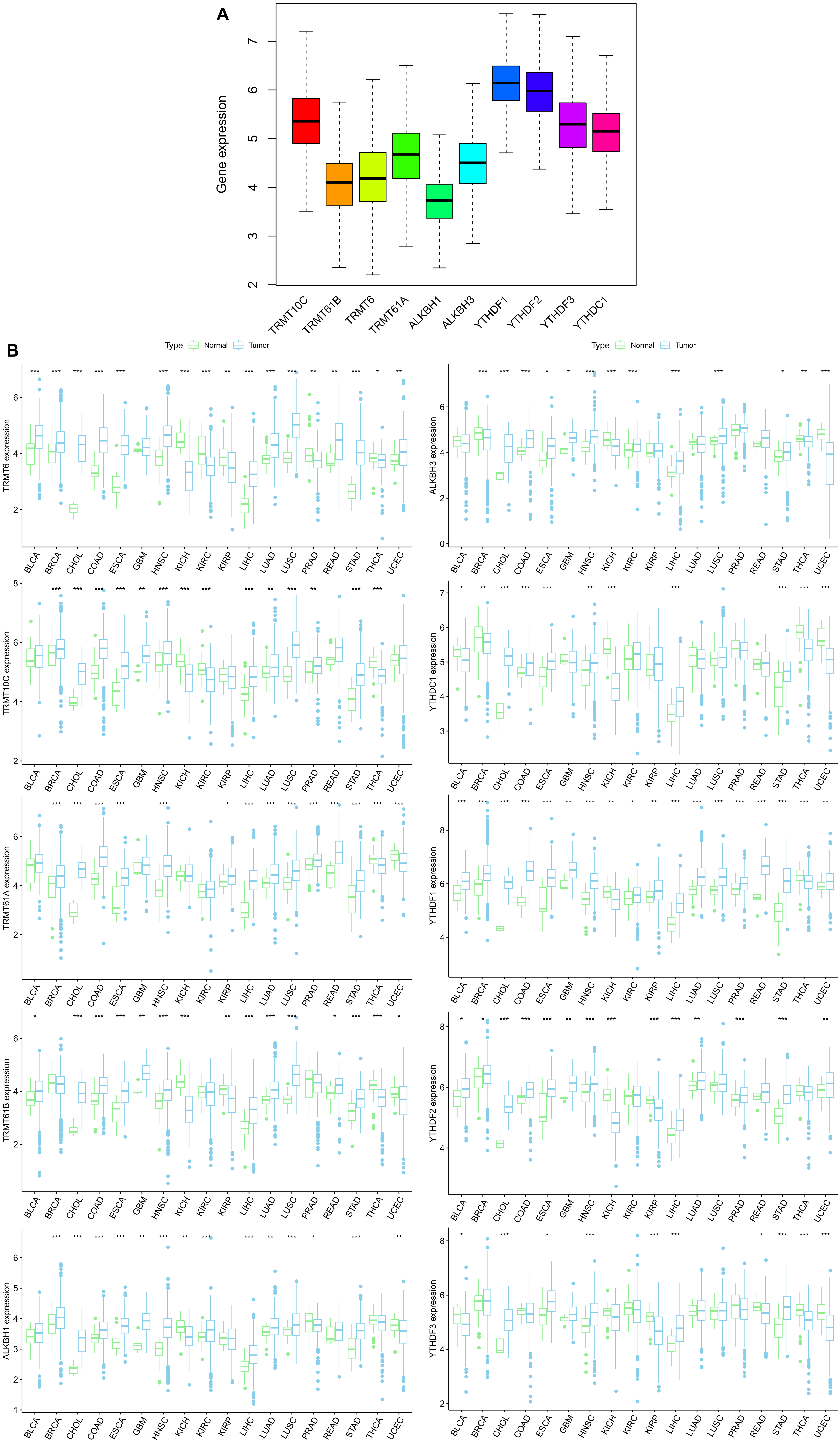

Figure S7. Expression levels of m1A regulators in TCGA cohort across pan-cancer analysis. (A) Expression distribution of m1A regulators in 33 cancer types. (B) Expression levels of m1A regulators in tumor tissues compared with the control tissues.
